# Telemedicine may increase visit completion rates in postpartum patients with preeclampsia

**DOI:** 10.1371/journal.pone.0275741

**Published:** 2022-10-21

**Authors:** Monika Sanghavi, Elizabeth Packard, Santina Sperling, Lauren A. Eberly, Marietta Ambrose, Howard M. Julien, Adi Hirshberg, Sri Adusumalli, Jennifer Lewey

**Affiliations:** 1 Department of Medicine, Division of Cardiology, Perelman School of Medicine at the University of Pennsylvania, Philadelphia, Pennsylvania, United States of America; 2 Department of Internal Medicine, University of Pennsylvania School of Medicine, Philadelphia, Pennsylvania, United States of America; 3 Division of Cardiology, University of Pittsburgh Medical Center Heart and Vascular Institute, Harrisburg, Pennsylvania, Pennsylvania, United States of America; 4 Penn Cardiovascular Outcomes, Quality, and Evaluative Research Center, Cardiovascular Institute, University of Pennsylvania, Philadelphia, Pennsylvania, United States of America; 5 Penn Cardiovascular Center for Health Equity and Social Justice, University of Pennsylvania, Philadelphia, Pennsylvania, United States of America; 6 Leonard Davis Institute of Health Economics, University of Pennsylvania, Philadelphia, Pennsylvania, United States of America; 7 Corporal Michael J. Crescenz VA Medical Center, Philadelphia, Pennsylvania, United States of America; 8 Department of Obstetrics and Gynecology, University of Pennsylvania, Perelman School of Medicine, Pennsylvania, United States of America; 9 CVS Health, Woonsocket, Rhode Island, United States of America; University of Oklahoma Health Sciences Center, UNITED STATES

## Abstract

Postpartum cardiovascular (CV) evaluation of women with preeclampsia is recommended to screen for and treat modifiable risk factors to reduce lifetime CV risk. However, attendance at in-person postpartum obstetric and cardiology clinic visits is low. The aim of this study was to compare the completion rate of new patient telemedicine visits to in-person office visits for patients with preeclampsia referred for postpartum hypertension management and CV risk assessment at a single center. There were 236 unique new patient visits scheduled during the study period. The average age was 30.3 years, 73.7% patients were Black, and 56.7% had Medicaid insurance. The completion rate was 32% for in-person clinic visits and 70% for telemedicine visits. Women who did not complete an office visit were more likely to be Black (87% vs. 56%, p < 0.01) and younger (29.1 vs. 31.4 years, p = 0.04) compared to those who completed a visit. Notably, this difference was not seen with telemedicine visits. Telemedicine may provide a novel opportunity to improve the care for blood pressure management and CV risk reduction in a vulnerable population at risk of premature CV disease.

## Introduction

Preeclampsia is an under recognized risk factor for cardiovascular disease (CV) in women and is associated with at least a 2-fold higher risk of chronic hypertension, ischemic heart disease, heart failure, and CV mortality [1]. Postpartum CV evaluation of women with preeclampsia is recommended to screen for and treat modifiable risk factors to reduce lifetime CV risk [2]. However, attendance at in-person postpartum obstetric and cardiology clinic visits is low. More than 40% of women do not attend the recommended postpartum visit [3], limiting the ability to provide effective counseling and increasing strain on the health care system. The clinical impact of this may be greater for Black women, who experience higher rates of preeclampsia and related morbidity, and more barriers to accessing prenatal and postpartum care. Telemedicine is an emerging tool that can improve access to care, reduce racial disparities, and improve outcomes across the health care spectrum. As a result of the coronavirus disease 2019 (COVID-19) pandemic, there was rapid adoption of telemedical care in 2020. A recent study showed that Black and female patients have higher rates of telemedicine completion for ambulatory care visits [4]. Our hypothesis is that telemedicine may be a more accessible channel of care for patients with preeclampsia seeking postpartum cardiovascular care.

The aim of this study was to compare the completion rate of new patient telemedicine visits to in-person office visits for patients with preeclampsia referred for postpartum hypertension management and CV risk assessment at the Hospital of the University of Pennsylvania (HUP).

## Methods

The University of Pennsylvania Institutional Review Board (IRB) determined that the project does not meet criteria for human subject’s research and therefore further IRB review was not required. This was determined by the Human Research Protections Program in the Office of the Institutional Review Board at the University of Pennsylvania.

Since 2018, patients who deliver at HUP and develop severe term preeclampsia requiring medications on discharge, preterm preeclampsia, or superimposed preeclampsia are scheduled for a postpartum visit in the women’s CV health clinic within 6 weeks of discharge. Using the electronic medical record, we extracted demographic information, visit type, and visit completion status for patients with preeclampsia with postpartum visits between March 1, 2020 to August 30, 2020 and from an identical time period in 2019 using computer-based extraction. Manual extraction was then used to abstract clinically relevant variables. This time frame was chosen due to the rapid uptake of telemedicine into clinical practice and an equivalent time period the year prior was used for comparison. From March 2020-June 2020, all women were offered a telemedicine visit due to strict COVID precautions in the outpatient clinic. Starting in July 2020, patients were given the option of in-person or telemedicine visit. Duplicate patient encounters were excluded. When visits were rescheduled, the completed visit or the last rescheduled visit was included. Self-reported race and ethnicity were recorded in the EMR. Data on median household income were obtained from the American Community Survey and linked to the patient’s zip code. Visit type and completion status were determined using billing data. Differences in patient characteristics and completion rates were compared using chi-squared test for categorical data and *t* tests for continuous data.

## Results

**Fig 1:**
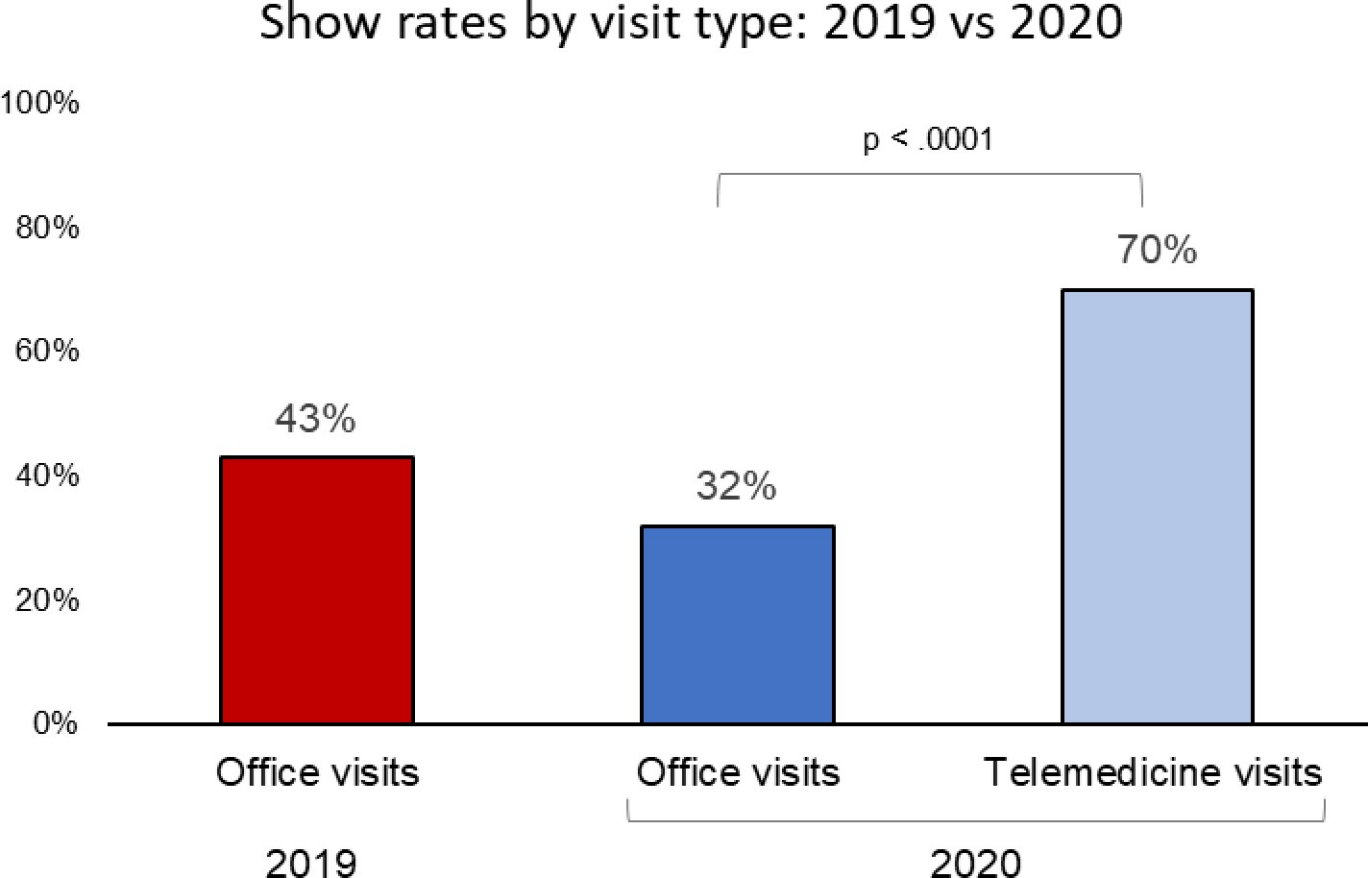
New patient visit completion rate by year and by visit type.

There were 236 unique new patient visits scheduled during the study period (117 visits in 2019 and 119 visits in 2020). The average age was 30.3 years, 73.7% patients were Black, and 56.7% had Medicaid insurance. Among 2019 in-person visits, the completion rate was 43%. Among 2020 clinic visits, the total completion rate was 44%. 40 visits (33.6%) were converted to telemedicine. The completion rate was 32% for in-person clinic visits and 70% for telemedicine visits (p < 0.001) (Fig 1). Although the proportion of telemedicine visits versus office visits decreased after July 2020, the show rate remained consistently higher for the telemedicine visits (S1 Fig).

Women who did not complete an office visit were more likely to be Black (87% vs. 56%, p < 0.01) and younger (29.1 vs. 31.4 years, p = 0.04) compared to those who completed a visit. Notably, this difference was not seen with telemedicine visits. There was no difference in show rate for office or telemedicine visits based on markers of disease severity (gestational age at delivery or antihypertensive prescription at discharge) or based on socioeconomic status (income or Medicaid insurance).

## Discussion

Our analysis suggests that telemedicine use in the postpartum setting for patients with preeclampsia is associated with higher visit completion rates for a population that traditionally has a high no-show rate. It is notable that the racial and age related differences seen for in-person visit completion was not present for telemedicine visits suggesting that coming to the clinic may be a greater barrier to care for some populations. Our Maternal Fetal Medicine colleagues have found novel ways to increase health care delivery and reduce disparities for this at-risk population through a text-based remote blood pressure monitoring program, minimizing the inconvenience of office-based follow-up [5]. The expansion of telemedicine to long-term postpartum hypertension management and future CV risk assessment may be another tool in improving the health care delivery to this vulnerable population and eliminating disparities in long-term CV health outcomes.

Our study has limitations. Our small sample size and single institution experience limits generalizability. Most patients with preeclampsia at HUP are discharged from the hospital with a home blood pressure monitor, which facilitates the remote monitoring of hypertension. Selection bias may be present among patients scheduled for a telemedicine visit; however, our analysis did not reveal significant differences between baseline characteristics in patients scheduled for a telemedicine versus in-person office visit in terms of socioeconomics or disease severity (Table 1). Although telemedical care has inherent limitations due to inability to examine the patient and obtain real time testing (EKG, echocardiogram), the postpartum preeclampsia visit is well suited for this technology since the visit is largely based on blood pressure management and preventive counseling, and the necessary vital signs can be obtained by the patient at home. Telemedicine approaches for blood pressure management have been shown to be effective and cost-effective [6].

**Table 1:** Baseline characteristics.

	2020 Visits: Entire Cohort (N = 119)			2020 Visits: Entire Cohort (N = 119)			2020 Office visit (N = 79)			2020 Telemed Visit (N = 40)		
	Tele med (N = 40)	Office Visit (N = 79)	p-value	Show (N = 53)	No show (N = 66)	p-value	Show (N = 25)	No show (N = 54)	p-value	Show (N = 28)	No show (N = 12)	p-value
**Age, years (mean, SD)**	30.8 (6)	30.3 (5.8)	0.28	31.2 (4.9)	29.7 (6.4)	0.09	31.4 (4.8)	29.1 (6.1)	0.04	30.5 (5.1)	31.3 (6.8)	0.15
**Race**												
**Black**	28 (70%)	61 (77%)	0.73	36 (68%)	53 (80%)	0.12	14 (56%)	47 (87%)	<0.01	22 (79%)	6 (50%)	0.07
**Hispanic**	1 (2.5%)	2 (2.5%)	0.99	1 (1.9%)	2 (3.0%)	0.69	1 (4.0%)	1 (1.9%)	0.57	0	1 (8.3%)	--
**Socio-economics**												
**Medicaid insurance**	20 (50%)	44 (56%)	0.56	24 (45%)	40 (61%)	0.10	11 (44%)	33 (61%)	0.15	13 (46%)	7 (58%)	0.49
**Median income <50,000**	28 (70%)	64 (81%)	0.18	39 (74%)	53 (80%)	0.39	19 (76%)	45 (83%)	0.44	20 (71%)	8 (67%)	0.76
**Disease Severity**												
**Delivery <37 weeks**	18 (45%)	41 (52%)	0.48	28 (53%)	33 (50%)	0.84	15 (60%)	26 (48%)	0.33	13 (46%)	7 (58%)	0.49
**Delivery <34 weeks**	6 (15%)	18 (23%)	0.32	13 (25%)	11 (17%)	0.29	9 (11%)	9 (17%)	0.06	4 (14%)	2(17%)	0.85
**Anti-HTN med prescribed on discharge**	20 (50%)	45 (57%)	0.47	27 (51%)	38 (58%)	0.47	13 (52%)	32 (59%)	0.54	14 (50%)	6 (50%)	1.00

Anti-HTN = antihypertensive; Telemed = Telemedicine

## Conclusions

The postpartum assessment of women with adverse pregnancy outcomes provides an important opportunity for primordial and primary prevention. Telemedicine may provide a novel opportunity to improve the care for blood pressure management and CV risk reduction in a vulnerable population at risk of premature CV disease. Payers and hospitals need to consider these factors when deciding on the reimbursement models and policies for telemedicine in the future.

## Supporting information

S1 Fig Show rate by visit type over time in 2020. (DOCX)

S1 File (XLSX)
