# Supplementary material for: Telemedicine may increase visit completion rates in postpartum patients with preeclampsia
Source: PLoS One. 2022 Oct 21;17(10):e0275741. doi: 10.1371/journal.pone.0275741 (PMC9586337; doi:10.1371/journal.pone.0275741)
Supplement: S1 Fig — (DOCX) [file pone.0275741.s001.docx]

**Figure S1: Show rate by visit type over time in 2020**


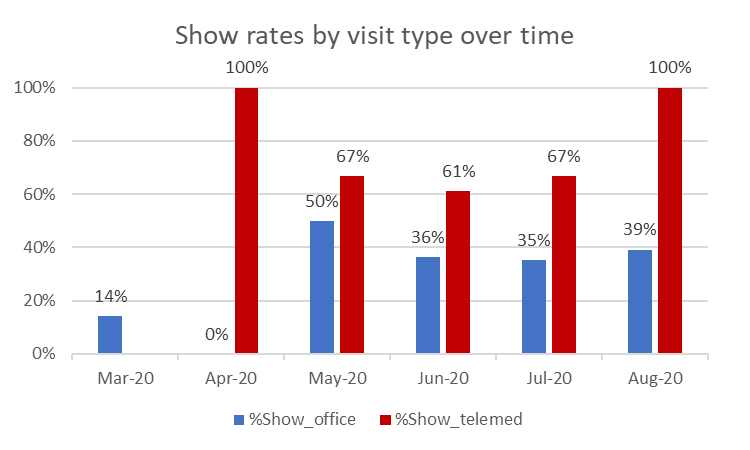


*Dotted line represents a change in clinic operations with a shift towards in-person visits
